# Ovariectomy in Canine Surgical Medicine: A Comparative Analysis of Surgical Approaches and the Nociceptive, Inflammatory, and Oxidative Stress Responses

**DOI:** 10.3390/ani15162336

**Published:** 2025-08-09

**Authors:** Giovanna Lucrezia Costa, Fabio Leonardi, Patrizia Licata, Martina Porcino, Federica De Paoli, Diego Iannelli, Fabio Bruno, Francesco Macrì, Nicola Maria Iannelli

**Affiliations:** 1 Department of Veterinary Sciences, University of Messina, Via Palatucci Annunziata, 98168 Messina, Italy; glcosta@unime.it (G.L.C.); plicata@unime.it (P.L.); fmacri@unime.it (F.M.); nicolamaria.iannelli@unime.it (N.M.I.); 2 Department of Veterinary Science, University of Parma, Via del Taglio 10, 43126 Parma, Italy; fabio.leonardi@unipr.it; 3 Clinica Veterinaria Camagna, Vetpartners, Via Fortunato Licandro 13, 84124 Reggio Calabria, Italy; federicadepaoli2@gmail.com (F.D.P.); diegoiannelli@hotmail.com (D.I.)

**Keywords:** canine ovariectomy, laparoscopy, oxidative stress, postoperative pain, inflammation, minimally invasive surgery, veterinary medicine

## Abstract

This study aimed to compare the physiological impact of open ovariectomy (the O group) and laparoscopic ovariectomy (the LA group) in dogs by evaluating intraoperative nociceptive responses, postoperative pain, inflammatory markers, and oxidative stress parameters. Sixty healthy dogs were randomly assigned to either technique or a control group (CNT). A standardized anesthetic protocol was used, and their vital signs, pain scores, and blood parameters were measured before, during, and after surgery. Pain was evaluated using the Colorado State University Canine Acute Pain Scale. Oxidative stress was measured by assessing levels of catalase (CAT), superoxide dismutase (SOD), myeloperoxidase (MPO), butyrylcholinesterase (BuChE), and malondialdehyde (MDA). Both techniques were safe and well tolerated, with no complications or need for rescue analgesia. However, the open surgery group exhibited significantly higher levels of inflammatory and oxidative stress markers postoperatively, including increased MPO and MDA levels and decreased BuChE. In contrast, the laparoscopic group showed only mild, transient changes, suggesting reduced tissue trauma. The higher glycemic levels observed in the open surgery group further suggest increased tissue trauma and systemic stress, reinforcing the conclusion that laparoscopy has a lower metabolic impact. Although the laparoscopic procedures require more time, their lower physiological impact may facilitate improved postoperative recovery. These results support laparoscopy as the preferred surgical approach in veterinary practice due to its association with improved metabolic stability and recovery outcomes.

## Simple Summary

Ovariectomy is routinely performed in female dogs in some regions of Europe to prevent reproduction and manage associated health conditions. This study compared two techniques: traditional open surgery and a newer method called laparoscopy, which uses small incisions and a camera. The aim was to determine which technique induces less perioperative pain and physiological stress in dogs. Sixty dogs were closely monitored throughout the preoperative, intraoperative, and postoperative periods. Physiological parameters such as heart rate, respiratory rate, and body temperature were assessed, and blood samples were collected to quantify inflammatory and oxidative stress biomarkers. All dogs were given the same anesthesia and pain relief. The results showed that both surgeries were safe and caused no serious complications. However, laparoscopy caused less stress and inflammation, with fewer changes in blood markers. The pain scores were minimal in both groups; however, the dogs undergoing laparoscopy exhibited reduced metabolic disturbances. Laparoscopic ovariectomy was associated with reduced physiological stress and inflammation compared to those after open surgery. These findings may assist veterinarians in selecting the most appropriate surgical approach.

## 1. Introduction

In some regions of Europe, ovariectomy is routinely performed in female dogs as a method of reproductive sterilization and health management. Traditionally, this procedure has been conducted using a conventional open laparotomy approach [1].

Although this method ensures effective removal of the ovaries, it is frequently accompanied by elevated postoperative discomfort, prolonged convalescence, and heightened physiological stress responses. In recent decades, the adoption of minimally invasive surgical techniques, such as laparoscopy, has become increasingly prevalent. These approaches are associated with several advantages, including reduced tissue disruption, diminished postoperative pain, and accelerated recovery times [2].

Surgical interventions like ovariectomy inherently involve multifactorial physiological and biochemical processes. Nevertheless, comprehensive studies focusing on perioperative and postoperative care protocols have only recently become the focus of systematic research [3,4]. While laparoscopic ovariectomy has gained increasing acceptance as a minimally invasive alternative to traditional open laparotomy, particularly in parts of Europe, a limited amount of comparative data remains regarding the physiological impact of these two surgical techniques in dogs. Specifically, although several studies have addressed aspects of surgical stress, postoperative pain, and recovery, few have systematically investigated the intra- and postoperative oxidative and nociceptive responses elicited by these procedures in a controlled setting.

Previous research has largely focused on clinical outcomes or broad physiological responses. However, specific biochemical markers of oxidative stress and inflammation have not been directly quantified. Moreover, although the interplay between oxidative stress and nociceptive signaling is recognized in other fields, it remains poorly characterized in veterinary surgical practice, especially during sterilization procedures in dogs.

In particular, data on the systemic metabolic alterations induced by surgical stress remain limited [5]. The physiological stress response during and after surgery is characterized by perturbations in homeostatic balance, reflected by altered plasma concentrations of catecholamines, glucagon, glucocorticoids, and glucose. These fluctuations are typically mediated by a range of stimuli, including oxidative injury and the systemic release of pro-inflammatory cytokines. Contributing factors to this stress cascade include surgical trauma, anesthetic agents, tissue hypoxia, perioperative hypothermia, dehydration, nociceptive input, blood loss, and microbial contamination [6,7,8].

A well-documented bidirectional relationship exists between oxidative stress and inflammation. Inflammatory responses are often accompanied by the increased production of reactive oxygen species (ROS), whereas anti-inflammatory pathways tend to reduce oxidative stress. Conversely, excessive ROS generation can activate inflammatory signaling pathways, while restoring redox balance may reduce inflammatory cell activity. This complex interaction plays a central role in the physiological response to surgical injury [9].

Oxidative stress can significantly affect enzymes involved in antioxidant defense, particularly superoxide dismutase (SOD) and catalase (CAT). Additionally, oxidative stress facilitates lipid peroxidation and amplifies inflammatory responses [10,11,12]. Malondialdehyde (MDA), a recognized marker of lipid peroxidation, is commonly used to assess oxidative damage in biomedical assays. MDA reacts with thiobarbituric acid (TBA) to form an MDA-TBA adduct, which shows peak absorbance at 532 nm, producing a characteristic pink-red hue. The thiobarbituric acid reactive substance (TBARS) assay is a widely accepted method for quantifying lipid peroxidation and evaluating oxidative stress in various biological matrices, including serum, low-density lipoproteins, and cellular lysates [13].

Despite the increasing use of laparoscopic ovariectomy, comparative data regarding the intra- and postoperative nociceptive responses and oxidative stress markers between laparotomy and laparoscopy in dogs are still scarce. Understanding these physiological responses is crucial for optimizing surgical approaches, improving animal welfare, and reducing postoperative complications.

This study aims to address this gap by providing a focused, comparative analysis of laparoscopic and open ovariectomy in female dogs, with particular emphasis on the intraoperative and early postoperative changes in oxidative stress biomarkers and nociceptive responses. By integrating biochemical, physiological, and surgical data, this study aims to clarify the systemic effects of these two techniques and support evidence-based improvements in veterinary surgical protocols that enhance animal welfare and clinical outcomes.

## 2. Materials and Methods

This study received ethical approval by the Review Board for Animal Care of the University of Parma, project no. 03/CESA/2023. and was conducted in strict accordance with the Italian regulations (DM 116192), European directives (Official Journal ECL 358/1, dated 18 December 1986), and United States legislation (Animal Welfare Assurance No. A5594-01, Department of Health and Human Services, Washington, DC, USA), specifically adhering to Legislative Decree No. 193 of 6 April 2006. Written informed consent was obtained from the owners of the dogs.

### 2.1. Sample Size Determination and the Study Design

The required sample size was calculated using G*Power software version 3.1. An a priori power analysis was conducted based on a one-way fixed-effects ANOVA (an omnibus test), assuming an effect size (f) of 0.45, a significance level (α) of 0.05, and a statistical power (1-β) of 0.80. The variance estimates were based on pilot data. This study employed a parallel-group experimental design with three independent groups.

A total of sixty healthy female dogs, aged 1.5 years ± 0.5 months and with a weight of 16 ± 0.5/Kg, were enrolled into this study. The animals were randomly assigned into two experimental groups, O and LA, using a lottery-based randomization method.

Group O underwent ovariectomy via a traditional open surgical approach (laparotomy), while the LA group underwent ovariectomy using a laparoscopic technique.

A control group (the CNT group), consisting of healthy female dogs, was recruited for the purpose of performing transvaginal ultrasonography to assess the presence of congenital abnormalities.

The sample size was determined a priori to provide 80% power to detect the specified effect size.

The control group (the CNT group) was established to provide baseline data on the impact of a non-invasive procedure involving the reproductive tract, such as transvaginal ultrasonography, on inflammatory and oxidative stress responses.

The primary inclusion criterion for the enrolment of subjects into this study was an indication for elective ovariectomy. Subjects were excluded if they exhibited abnormalities in their complete blood count or serum biochemical profile or if they presented with ovarian cysts, inflammatory conditions, or active neoplastic disease.

### 2.2. The Anesthetic Protocol and Pharmacological Management

The dogs were fed dry food once daily and had unrestricted access to water until at least eight hours before the induction of anesthesia. Preanesthetic medications included a subcutaneous injection of meloxicam at a dosage of 0.2 mg/kg (Metacam 2%, Boehringer Ingelheim Italia S.p.A., Milan, Italy) and an intramuscular injection of atropine sulfate at 0.03 mg/kg as an anticholinergic (Atropine sulfate 0.1%, A.T.I. Bologna, Italy). A 20-gauge, 32 mm intravenous catheter (DELTA VEN) was placed into the cephalic vein to facilitate the continuous administration of lactated Ringer’s solution at a rate of 5 mL/kg/h throughout the surgical intervention.

Anesthesia was induced intravenously 20 min after premedication with propofol at a dose of 4 mg/kg (Proposure 1%, Merial, Assago, Italy). Following induction, endotracheal intubation was performed using a cuffed Magill-type tube. Anesthesia was maintained using sevoflurane (Sevoflo, Zoetis, Catania, Italy) delivered in 100% oxygen via a rebreathing circuit. Mechanical ventilation was applied using a pressure-controlled ventilator (SIMV, GE Datex-Ohmeda Avance, Ultramed Srl, Catania, Italy) with the following parameters: the respiratory rate set at 12 breaths per minute, a positive end-expiratory pressure (PEEP) of 4 cm H_2_O, an inspiratory-to-expiratory (I:E) ratio of 1:7, and a peak airway pressure of 12 cm H_2_O. Postoperative analgesia consisted of oral meloxicam administered at 0.1 mg/kg once every 24 h.

### 2.3. Monitoring of Vital and Physiological Parameters

The following physiological parameters were continuously monitored throughout the surgical procedure—heart rate (HR, beats per minute), assessed initially through cardiac auscultation using a Littmann stethoscope (USA) and subsequently via the anesthesia monitor after induction; the respiratory rate (RR, breaths per minute), measured through a visual assessment of thoracic excursions at the baseline and monitored electronically thereafter; non-invasive blood pressure, including the systolic (SAP), mean (MAP), and diastolic (DAP) values, measured using a cuff placed at the base of the tail; body temperature (T, °C); end-tidal carbon dioxide concentration (EtCO_2_, mmHg); arterial oxygen saturation (SpO_2_, %); and both inspired (CSI) and expired concentrations (CES) of sevoflurane (FiSevo and EtSevo)—with all recorded using a multiparametric anesthesia monitoring system (GE Datex-Ohmeda Avance, Ultramed Italy).

Measurements were recorded at the following time points: the baseline (T0), following a 30 min acclimation period in the surgical preparation room; 20 min post-premedication with meloxicam and atropine (excluding EtCO_2_ and sevoflurane concentrations); immediately after the induction of anesthesia; at skin incision; during laparotomy; during traction and the removal of each ovary; and at the completion of skin suturing.

### 2.4. Evaluation of Intraoperative and Postoperative Nociceptive Responses

The intraoperative nociceptive response to surgical stimuli was evaluated using a composite pain score adapted from Costa et al., 2022 [14], with a ≥20% increase in the HR, RR, or SAP considered indicative of nociceptive stimulation. An excessive surgical stimulus may lead the patient to oppose the mechanical ventilator, potentially resulting in increased respiratory rates compared to the preset values. When this threshold was exceeded, rescue analgesia was administered, consisting of intravenous fentanyl at 2 μg/kg (Fentadon, Dechra).

Postoperative pain was assessed using the Colorado State University Canine Acute Pain Scale, ranging from 0 (no pain) to 4 (severe pain), at 6 h intervals for 24 h post-surgery. Scores were assigned by three independent observers who were blinded to the surgical technique to which the patients had been subjected. A score of ≥2 (mild to moderate pain) triggered the administration of postoperative rescue analgesia, which consisted of 0.2 mg/kg of methadone (Semfortan, Dechra) given via intramuscular injection.

### 2.5. Blood Sample Collection

Blood samples were collected following the assessment and documentation of the physiological parameters at the baseline and at 12, 24, and 48 h post-surgery/ultrasonography. A volume of 5 mL of venous blood was drawn from the cephalic vein by a single trained operator to ensure procedural consistency. The collected blood was divided into two portions: one aliquot was transferred into a vacuum serum separator tube containing a clot activator (Vacuette^®^, Greiner Bio-One, Kremsmünster, Austria) for the determination of the biochemical parameters, antioxidant enzyme activities, and lipid peroxidation markers. The second portion was placed into a tube containing K3-EDTA (Vacuette^®^, Greiner Bio-One) for hematological examination, which was performed exclusively at the baseline. The serum samples were immediately stored at 4 °C and centrifuged within 3 to 4 h post-collection at 1500× *g* for 15 min to obtain clarified serum for the subsequent analyses.

### 2.6. Assessment of the Hematological and Biochemical Parameters

A complete blood count (CBC) was performed exclusively at the baseline using the standard hematological techniques. Both serum and EDTA-treated whole blood samples were maintained at 4 °C immediately following collection. Serum was separated through centrifugation at 1500× *g* for 15 min. All spectrophotometric measurements were carried out using a UV–visible spectrophotometer (A560).

The biochemical parameters evaluated included glucose, aspartate aminotransferase (AST), alanine aminotransferase (ALT), total protein, albumin, and blood urea nitrogen (BUN). These parameters were measured at the baseline and 12 h post-surgery/ultrasonography. Glucose levels were determined using the glucose oxidase–peroxidase enzymatic method; total protein through the biuret assay; and albumin using the bromocresol green method, and AST and ALT activities were assessed via kinetic enzymatic assays conducted at 37 °C.

### 2.7. The Evaluation of Lipid Peroxidation Markers

The antioxidant enzyme activities determined included catalase (CAT), superoxide dismutase (SOD), myeloperoxidase (MPO), and butyrylcholinesterase (BuChE). Catalase (CAT) activity was quantified based on its ability to catalyze the decomposition of hydrogen peroxide (H_2_O_2_). Prior to the enzymatic assay, all reagents were equilibrated to ambient temperature. Each well of the microplate contained a final volume of 240 µL. Both the samples and standards were analyzed in duplicate using a 1× catalase sample buffer along with a diluted assay buffer. Absorbance readings were obtained at 540 nm using a BIORAD 680 microplate reader (Bio-Rad Laboratories Srl, Milan, Italy).

Superoxide dismutase (SOD), a class of metalloenzymes that catalyze the dismutation of superoxide radicals into molecular oxygen and hydrogen peroxide, was also evaluated. The final reaction volume per well was 230 µL. The reagents were equilibrated to 25 °C prior to assay initiation, except for the xanthine oxidase enzyme. The absorbance was recorded between 440 and 460 nm using the same plate reader.

Myeloperoxidase (MPO) activity was assessed via the o-dianisidine-H_2_O_2_ assay in a 96-well format. Triplicate samples were added to a reagent mixture comprising 0.53 mM o-dianisidine hydrochloride, 0.15 mM hydrogen peroxide, and 50 mM potassium phosphate buffer at a pH o 6.0. After a 5 min incubation at 25 °C, the enzymatic reaction was terminated with 30% sodium azide, and the absorbance was read at 460 nm using the BIORAD 680 microplate reader.

Butyrylcholinesterase (BuChE) activity was determined through the hydrolysis of a thiocholine substrate, which reacts with 2-nitrobenzoic acid in 625 µL of BuChE assay buffer to form a colored product. The absorbance was immediately measured at 412 nm at 25 °C using the BIORAD 680 reader.

### 2.8. Quantification of Lipid Peroxidation via Malondialdehyde (MDA)

Lipid peroxidation markers were quantified using malondialdehyde (MDA).

All of the reagents used, including phosphoric acid (85%, 15 mol/L), sodium hydroxide, sodium dodecyl sulfate (SDS, 8.1%), and sodium chloride, were obtained from Merck (Darmstadt, Germany). Thiobarbituric acid (TBA) was sourced from Fluka (Buchs, Switzerland), and all of the chemicals were of analytical grade or the highest available purity.

The malondialdehyde (MDA) standards were prepared via acid hydrolysis of 1,1,3,3-tetramethoxypropane (TMP). The TBA reagent (0.11 mol/L) was freshly prepared by dissolving 800 mg of TBA into 50 mL of 0.1 mol/L NaOH. For the thiobarbituric acid reactive substance (TBARS) assay, 200 µL of MDA standard solution was used in place of plasma. TMP (10 mmol/L, 50 µL) was hydrolyzed in 10 mL of 0.01 mol/L HCl for 10 min at room temperature to generate the MDA stock solutions, which were then diluted in ultrapure water to obtain a standard curve with varying MDA concentrations.

Calibration in serum was achieved by supplementing pooled plasma samples with phosphoric acid containing known MDA concentrations. The sample aliquots (200 µL) were incubated with the reaction mixture at 90 °C in a water bath for 1 h to induce the reaction between MDA and TBA in acidic conditions. The reactions were halted by placing the tubes on ice [15]. Following cooling, 100 µL of each reaction mixture was transferred into a flat-bottom 96-well plate. The absorbance was measured at 535 nm and 572 nm to correct for the background absorbance. The MDA levels (expressed as TBARS equivalents) were calculated from the differential absorbance using a standard calibration curve.

### 2.9. The Surgical Technique

All of the animals underwent an abdominal trichotomy, followed by alternate-step antisepsis of the surgical area using a chlorhexidine–alcohol solution to ensure optimal skin asepsis.

#### 2.9.1. Open Ovariectomy (The O Group)

In the open surgery group, the dogs were positioned in dorsal recumbency. A midline laparotomy was performed via a 4 cm linear incision, allowing for direct access to the peritoneal cavity. Upon identification of the right ovary, the proper ovarian ligament was atraumatically stretched, and the caudal pole of the ovary, including the uterine tube, was ligated using a 0-gauge braided absorbable polyglyconate suture (Monosyn, B. Braun) [16]. The ovariectomy was then completed through careful dissection of the surrounding connective tissue and transection of the vascular pedicle between the two pre-placed ligatures. An identical technique was applied to the excision of the contralateral ovary.

Abdominal wall closure was performed in three anatomical layers. The muscular fascia was closed using a continuous suture pattern with interspersed simple interrupted stitches, employing a 0-gauge braided absorbable suture. The subcutaneous layer was closed using a simple continuous pattern with 2-0 braided absorbable polyglyconate suture material (Monosyn, B. Braun). The intradermal layer was approximated with the same suture type and gauge as the subcutaneous layer, using a buried continuous intradermal technique.

#### 2.9.2. Laparoscopic Ovariectomy (The LA Group)

Dogs assigned into the laparoscopic group underwent an ovariectomy using a standardized three-port technique, as described by Bendinelli et al., 2019 [17]. The subjects were placed in dorsal recumbency, with the surgical table adjusted to a slight Trendelenburg or reverse Trendelenburg tilt as required to optimize organ visualization [18]. All procedures were performed by the same experienced surgeon to ensure consistency.

A 10 mm trocar (T1; MedLine, Florence, Italy) was introduced at the umbilical region on the ventral midline to establish pneumoperitoneum using carbon dioxide. The intra-abdominal pressure was maintained at 12 mmHg via an automated insufflation device (Karl Storz Endoscopy, Rome, Italy). Visualization was achieved using a 30°, 10 mm, 31 cm laparoscope (Karl Storz Endoscopy, Italy). A 5 mm trocar (T2; MedLine) was placed between the umbilicus and the xiphoid process, and an additional 10 mm trocar (T3; MedLine) was inserted midway between the umbilicus and the pubis.

Each ovary was identified and atraumatically grasped at the proper ovarian ligament using endoscopic forceps (Wolf Medical Instruments, Vernon Hills, USA). Subsequently, the ovarian pedicles were coagulated and transected using a bipolar vessel sealing device (PL720SU-Caiman, Aesculap, B. Braun). Both ovaries were exteriorized through the T2 port. Upon completion of the procedure, the pneumoperitoneum was released via gentle manual abdominal compression, and all trocars were removed. The umbilical port site, including the fascia, external abdominal oblique muscle, and subcutaneous tissue, was closed in separate layers using 2-0 polyglyconate suture material (Monosyn, B. Braun) in a simple continuous pattern. The skin was sealed using a topical tissue adhesive (Vet Bros Company, Perugia, Italy).

#### 2.9.3. Surgical Timing and Complication Assessment

The total operative time was defined as the period from the initial skin incision to final skin closure. In addition, the duration required for the excision of each ovary was recorded separately. In the laparoscopic group, the timing for ovarian removal began upon the insertion of the grasping forceps through port T2. In the open surgery group, timing commenced at the placement of the initial ligature around the ovarian pedicle.

All intraoperative and postoperative complications were systematically recorded and classified. Major intraoperative complications were defined as events requiring significant deviation from the standardized surgical protocol (e.g., emergency conversion into open surgery, inadvertent visceral injury, or uncontrolled hemorrhage). Minor intraoperative complications were those that were managed without procedural deviation (e.g., minor bleeding, suture line disruption).

Postoperative complications were similarly categorized. Major postoperative complications included events necessitating further veterinary intervention (e.g., surgical site infection, persistent seroma). Minor complications were defined as self-limiting and not requiring therapeutic intervention (e.g., localized bruising, erythema, swelling, or subcutaneous emphysema at the incision sites) [19,20].

## 3. The Statistical Analysis

The data analysis was performed using SPSS software, version 27.1 (IBM Corp., Novegro-Tregarezzo, Italy). The Shapiro–Wilk test was applied to assessing the normality of the data distribution, and the results were presented either as the mean ± standard deviation (SD) or as the median with the range (score). Changes in CAT, SOD, MPO, BuChE, MDA, and biochemical parameters over time and between groups were analyzed using a two-way repeated measures analysis of variance (ANOVA), followed by Bonferroni post hoc correction for multiple comparisons. Differences in the surgical durations between operative techniques were evaluated using an independent samples *t*-test.

Differences in scores were evaluated using a Wilcoxon–Mann–Whitney test. Inter-observer reliability for postoperative pain assessments was determined using Kendall’s coefficient of concordance (W). SPSS automatically applies base-10 logarithmic transformation for normalization purposes. A *p*-value of <0.05 was considered indicative of statistical significance.

## 4. Results

A total of 60 dogs were enrolled in this study, yielding a statistical power of 0.80. The data distribution did not conform to normality. The inter-observer agreement for the postoperative pain evaluation using the Canine Acute Pain Scale was perfect (W = 1). All enrolled animals completed the study protocol.

The physiological parameters remained within the expected ranges for animals under general anesthesia. No subject exhibited an increase greater than 20% in its heart rate (HR), respiratory rate (RR), or systolic arterial pressure (SAP) during surgery. The oxygen saturation (SpO_2_) ranged between 96% and 100%, indicating sufficient peripheral oxygenation. End-tidal carbon dioxide (ETCO_2_) levels gradually decreased from 43 mmHg to between 32 and 36 mmHg, suggesting optimal ventilator adaptation and a stable anesthetic depth. Sevoflurane concentrations were maintained within consistent ranges throughout: 3–4% inspired and 3–6% expired.

Postoperative pain scores remained at 0 throughout the observation period in all dogs, indicating the absence of clinically detectable pain. No subject required intraoperative or postoperative rescue analgesia. Moreover, no surgical or anesthetic-related complications were observed.

The mean duration required for bilateral ovariectomy was significantly different between groups: 45 ± 0.5 min in the laparoscopic group and 10 ± 0.2 min in the open surgery group *p* = 0.000. See Table 1.

Table 2 presents the temporal profiles of antioxidant and oxidative stress biomarkers, including catalase (CAT), superoxide dismutase (SOD), myeloperoxidase (MPO), butyrylcholinesterase (BuChE), and malondialdehyde (MDA), at the baseline (B) and 24 h and 48 h post-surgery across three experimental groups: the control (CNT) group, the O group, and the LA group. Catalase (CAT) activity remained stable over time in the CNT group. In the O group, a significant increase was observed at 24 h (9.5 ± 0.2 U/mL) compared to that at the baseline (8.3 ± 0.4 U/mL, *p* < 0.05), followed by a significant decrease at 48 h (7.5 ± 0.3 U/mL). The LA group also exhibited significant elevation at 24 h (9.4 ± 0.2 U/mL, *p* < 0.05), returning to near-baseline levels by 48 h. These findings suggest the transient induction of catalase postoperatively, particularly in the treated groups.

Superoxide dismutase (SOD) levels in the CNT group remained largely unchanged. The O group showed a significant increase at 24 h (2.8 ± 0.03 U/mL, *p* < 0.05), followed by a decrease at 48 h (2.2 ± 0.02 U/mL), while the LA group demonstrated a similar transient rise at 24 h (2.6 ± 0.04 U/mL, *p* < 0.05). These changes may reflect an acute oxidative stress response with subsequent normalization.

The myeloperoxidase (MPO) activity did not fluctuate significantly in the CNT group. The O group showed a marked increases at both 24 h (1.60 ± 0.05 U/mL, *p* < 0.05) and 48 h (1.55 ± 0.02 U/mL, *p* < 0.05) compared to that at the baseline. The LA group, however, did not show significant changes, indicating a potentially different inflammatory response between treatments.

Butyrylcholinesterase (BuChE) levels in the CNT group slightly increased over time. In the O group, a significant reduction was observed at 24 h (0.35 ± 0.2 U/mL, *p* < 0.05) and remained suppressed at 48 h. The LA group exhibited minimal variation. The suppression of BuChE in the O group may reflect cholinergic dysfunction or increased systemic inflammation.

Malondialdehyde (MDA) levels, indicative of lipid peroxidation, were stable in the CNT group and the LA group. In contrast, the O group exhibited a significant increase at 24 h (70 ± 3 μg/mL, *p* < 0.05 vs. baseline and vs. CNT), followed by a partial reduction at 48 h (56 ± 2.5 μg/mL, still significantly elevated compared to the baseline). This suggests a pronounced oxidative stress response in the O group, which was attenuated but still evident at 48 h. The LA group did not significantly differ from the control, indicating potential protective effects. See Table 2.

A statistically significant increase in blood glucose levels was observed 12 h after surgery in all groups (*p* < 0.05), including the control group. This likely reflects a physiological stress response to anesthesia, surgical manipulation, or handling.

At the baseline, significant differences in glycemia were noted between CNT and LA (β) and between O and LA (γ), suggesting pre-existing metabolic variability among groups.

Postoperatively, the O group exhibited the highest glycemic elevation (from 4.9 to 6.9 mmol/L), possibly indicating a more intense stress response associated with the open surgical approach. A significant decrease in albumin concentrations was observed 12 h after surgery in all groups (*p* < 0.05), with the most marked reduction occurring in the O group (from 6.5 to 3.2 g/dL). This reduction may be attributed to acute-phase response mechanisms, fluid shifts, or hemodilution, all commonly associated with surgical stress and perioperative fluid therapy.

No significant changes were detected in the ALT and AST values across time points or between groups. This suggests no evidence of acute hepatocellular injury, and both surgical approaches appear to be safe with respect to hepatic function in the immediate postoperative period. The total protein and BUN levels remained stable between the baseline and 12 h post-surgery in all groups, indicating no significant protein loss or catabolism and preserved renal function, with no evidence of dehydration or renal impairment in the short-term postoperative window. Both surgical techniques (open and laparoscopic ovariectomy) are well tolerated from a metabolic standpoint. The transient hyperglycemia and hypoalbuminemia observed postoperatively are likely part of a normal stress and inflammatory response and not indicative of complications. The greater albumin drop and glycemic rise in the open surgery group may reflect a higher degree of tissue trauma and systemic response compared to those under laparoscopy, potentially favoring the minimally invasive approach in terms of its metabolic impact. See Table 3.

## 5. Discussion

This study provides a comprehensive comparative analysis of the nociceptive, inflammatory, and oxidative stress responses associated with traditional open laparotomy (the O group) versus minimally invasive laparoscopic ovariectomy (the LA group) in dogs, alongside a control group (CNT). The operative time was significantly longer in the laparoscopic group (mean: 45 min) compared to that for open surgery (mean: 10 min), consistent with the technical demands and the learning curve associated with minimally invasive procedures [21]. Although the extended duration did not result in significant intraoperative physiological changes—heart rate, respiratory rate, and systolic arterial pressure remained stable across groups—this prolonged anesthesia time warrants further consideration [22,23]. An extended operative time can increase the duration of exposure to anesthetic agents, potentially affecting cardiopulmonary stability, thermoregulation, and postoperative recovery, particularly in high-risk or geriatric patients.

Moreover, longer surgical times may have economic implications. An increased anesthesia duration contributes to higher costs related to anesthetic drugs, monitoring, personnel time, and equipment usage. This factor may limit the widespread adoption of laparoscopic procedures, particularly in general veterinary practices or facilities with restricted access to laparoscopic instrumentation and trained personnel.

Thus, while the minimally invasive approach offers advantages in terms of reduced tissue trauma, attenuated inflammatory responses, and negligible postoperative pain, these benefits must be weighed against the operational and logistical challenges posed by longer surgical times. Thus, the choice to implement a laparoscopic ovariectomy should take into account not only physiological outcomes but also institutional resources, surgical expertise, and the appropriate patient selection, ensuring a balanced and context-specific application.

Our findings contribute valuable insights into the physiological impacts of these surgical approaches and their implications for clinical practice in veterinary surgical medicine. In the present study, two distinct surgical techniques for canine ovariectomy (open ovariectomy (the O group) and laparoscopic ovariectomy (the LA group)) were employed and analyzed, with specific attention to the surgical approach, tissue handling, and perioperative outcomes. A key methodological element was the atraumatic handling of the ovarian suspensory ligament, which was preserved intact in both procedures, in accordance with the best-practice guidelines described by Shivley et al. (2018) [16]. This approach is significant, as avulsion or forcible tearing of the ovarian ligament, although traditionally practiced, has been associated with increased perioperative pain and a heightened risk of hemorrhage and iatrogenic trauma to the surrounding tissues.

In the open ovariectomy group, the proper ovarian ligament was directly ligated using a 0-gauge braided absorbable suture (Monosyn, B. Braun), followed by careful dissection of the surrounding connective tissue. The ovarian pedicle was then transected between two secure ligatures. Importantly, the suspensory ligament was not subjected to blunt tearing but was instead managed through sharp dissection under direct visualization. This technique facilitated atraumatic excision of the ovary while minimizing tissue disruption and ensuring hemostatic control.

Similarly, in the laparoscopic group, the ovary was atraumatically grasped at the level of the proper ligament using endoscopic forceps. The ovarian pedicle was then safely coagulated and transected using a bipolar vessel sealing device (Caiman, Aesculap, B. Braun), without applying traction or mechanical tearing forces to the suspensory ligament. Precise energy-based sealing of the vascular structures minimized thermal spread and ensured effective hemostasis, thereby reducing intraoperative risk.

The avoidance of ovarian suspensory ligament avulsion in both groups is particularly significant, particularly given growing evidence linking ligament tearing with elevated pain scores postoperatively, as well as the potential for hemorrhagic complications [9]. Instead, the controlled handling and dissection observed in both the open and laparoscopic groups reflect a refined surgical approach aiming to optimize animal welfare and procedural safety.

Furthermore, standardization of the technique across individuals, including the consistent performance by a single experienced surgeon in the laparoscopic group, minimized the procedural variability and reinforced the validity of the comparative outcomes between the two methods.

In conclusion, both surgical techniques adhered to the current recommendations advocating for non-traumatic management of the ovarian ligament, thereby reducing intraoperative trauma and potentially contributing to the low complication rates observed in this study. These findings underscore the importance of gentle tissue handling and the avoidance of ligament avulsion in achieving favorable surgical and postoperative outcomes in canine ovariectomy.

Postoperative hyperglycemia was observed in all groups, including the controls, likely reflecting a nonspecific stress response to anesthesia and perioperative handling. Notably, the open surgery group exhibited the highest glycemic rise, which may signify a more pronounced systemic stress and inflammatory reaction due to the greater tissue trauma inherent in laparotomy. This aligns with the existing literature documenting stress-induced hyperglycemia as a marker of the severity of surgical insult [22,24].

Albumin levels declined significantly post-surgery in all groups, with the most substantial decrease in the O group. Hypoalbuminemia is a recognized acute-phase response to surgical stress, attributable to increased vascular permeability, fluid shifts, and possibly hemodilution due to perioperative fluid administration. The greater albumin reduction in the open surgery cohort further supports the conclusion that open ovariectomy elicits a more intense inflammatory and metabolic disturbance relative to that under laparoscopy [25].

No significant alterations were observed in the hepatic enzymes (ALT, AST) or renal markers (BUN), indicating that neither surgical technique caused acute hepatic or renal dysfunction during the immediate postoperative period. Stable total protein levels reinforce the conclusion that no significant protein catabolism or systemic loss occurred, emphasizing the metabolic tolerance of both procedures [25].

Oxidative stress markers revealed differential responses between the two surgical groups. Both the catalase (CAT) and superoxide dismutase (SOD) activities increased transiently 24 h post-surgery in the O and LA groups, reflecting an acute induction of endogenous antioxidant defenses in response to surgical insult. This transient rise likely represents a physiological adaptation to increased reactive oxygen species (ROS) generation [26]. Importantly, myeloperoxidase (MPO), an enzyme linked to neutrophil activation and inflammatory responses, was significantly elevated only in the open surgery group, indicating heightened inflammatory activation compared to that in the laparoscopic group [27]. Similarly, butyrylcholinesterase (BuChE) levels were suppressed in the O group, which may be indicative of cholinergic pathway perturbation linked to systemic inflammation [28].

Malondialdehyde (MDA) levels, a reliable indicator of lipid peroxidation and oxidative damage, were markedly elevated 24 and 48 h postoperatively in the open surgery group, whereas these levels in the laparoscopic and control groups remained stable. This finding highlights that open ovariectomy induces a more pronounced oxidative stress response, which may contribute to postoperative complications and slower recovery [13,26,29]. Collectively, these data reinforce the notion of a bidirectional relationship between inflammation and oxidative stress, where increased ROS production following open surgery potentiates inflammatory cascades, while laparoscopic surgery appears to mitigate this response, likely due to reduced tissue trauma and ischemia–reperfusion injury [30]. Our results suggest that laparoscopic ovariectomy in dogs offers a superior profile concerning metabolic, inflammatory, and oxidative stress parameters compared to that under traditional open surgery [31]. The minimal disruption of physiological homeostasis observed with laparoscopy, as indicated by oxidative stress markers, blood glucose, and albumin levels, supports its potential as a less physiologically invasive technique.

Nonetheless, the longer operative time for laparoscopy should be balanced against these benefits, especially considering the requirement for specialized equipment and surgical expertise [32]. Additional longitudinal studies are needed to evaluate the long-term outcomes, including chronic pain, the rate of recovery, delayed postoperative complications, and cost effectiveness in routine veterinary practice

## 6. Conclusions

In conclusion, both surgical techniques have proven to be safe and effective for canine ovariectomy. However, laparoscopy appears to be associated with a reduction in systemic inflammatory responses and oxidative stress during the postoperative period. These findings highlight the differences in metabolic and biochemical markers between the two procedures, providing a foundation for further studies aiming to determine whether these variations translate into tangible clinical benefits. Additional research is needed to assess the long-term impact of these techniques on recovery and overall animal welfare.

## Figures and Tables

**Table 1:** Colorado pain scores after postoperative analgesia was assigned, from awakening every 6 h to 24 h after surgery (scores from 0 to 4). Point 2 (moderate and mild pain) was the cut-off point for the administration of postoperative rescue analgesia.

CSU-CAPS					
	Awakening	6 h	12 h	18 h	24 h
Group O	0(0/0)	1(0/1) *	1(0/1) *	1(1/1) *	1(1/2) *
Group LA	0(0/0)	0(0/1) *	1(0/1) *	1(0/1) *	1(1/1) *

* Data on the difference along the timeline expressed as the median and range.

**Table 2:** Malondialdehyde (MDA), catalase (CAT), superoxide dismutase (SOD), myeloperoxidase (MPO), and butyrylcholinesterase (BuChE) at the baseline (B) and 24 h and 48 h after surgery/ultrasonography in the O, LA, and CNT groups.

	Baseline	24 h After Surgery/ Ultrasonography	28 h After Surgery/ Ultrasonography
CAT (U/mL)			
CNT	8.4 ± 1.2	8.4 ± 1.1	8.39 ± 1.1
O	8.3 ± 0.4	9.5 ± 0.2 *	7.5 ± 0.3 *
LA	8.5 ± 0.6	9.4 ± 0.2 *	8.3 ± 0.3
SOD (U/mL)			
CNT	2.3 ± 0.2	2.3 ± 0.3	2.4 ± 0.5
O	2.5 ± 0.06	2.8 ± 0.03 *	2.2 ± 0.02
LA	2.4 ± 0.3	2.6 ± 0.04 *	2.4 ± 0.04
MPO (U/mL)			
CNT	1.50 ± 0.06	1.51 ± 0.05	1.48 ± 0.05
O	1.45 ± 0.06	160 ± 0.05 *	1.55 ± 0.02 *
LA	1.47 ± 0.01	150 ± 0.08	1.46 ± 0.05
BuChE (U/mL)			
CNT	0.57 ± 0.3	0.60 ± 0.3	0.62 ± 0.23
O	0.56 ± 0.2	0.35 ± 0.2 *	0.40 ± 0.2 *
LA	0.57 ± 0.07	0.55 ± 0.1	0.54 ± 0.08
MDA (μg/mL)			
CNT	54 ± 1.5	53.8 ± 1.4	54.2 ± 1.2
O	53.7 ± 1.9	70 ± 3 *	56 ± 2.5 *
LA	54 ± 1.4	55 ± 1.5	54 ± 1.8

* Differences from the baseline. The values are expressed as the mean ± SD.

**Table 3:** Biochemical parameters evaluated: glycemia, aspartate aminotransferase (AST), alanine aminotransferase (ALT), total protein, albumin, and blood urea nitrogen (BUN). These parameters were measured at the baseline and 12 h after surgery and ultrasonography in the O, LA, and CNT groups.

	CNT		O		LA	
	Baseline	12 h After Surgery/ Ultrasonography	Baseline	12 h After Surgery/ Ultrasonography	Baseline	12 h After Surgery/ Ultrasonography
Glycemia (mmol/L)	5 ± 1.2	5.3 ± 3.4 *	4.9 ± 1.8	6.9 ± 1.2 * ^α^	6 ± 2.1	6.5 ± 1.3 * ^β γ^
Albumin (g/dL)	5 ± 0.2	4.5 ± 0.3 *	6.5 ± 0.5	3.2 ± 0.3 *	6 ± 0.7	5.2 ± 0.1 *
ALT (U/L)	17 ± 2	17 ± 2.4	19.5 ± 0.5	20 ± 0.2	20 ± 0.3	21.2 ± 0.5
AST (U/L)	27.5 ± 6.3	28 ± 6.5	32 ± 0.6	32.5 ± 0.3	20.5 ± 2.1	21 ± 0.5
Total Protein (g/dL)	6.6 ± 0.2	6.6 ± 0.4	6.4 ± 0.3	6.5 ± 0.1	6.3 ± 0.1	6.3 ± 0.4
BUN (mg/dL)	18 ± 1.5	18 ± 1.3	23 ± 1.4	23 ± 1.2	21 ± 1.5	21.5 ± 0.5

* Differences from baseline; ^α^ difference between groups CNT and O; ^β^ difference between groups CNT and LA; ^γ^ difference between groups O and LA. The values are expressed as the mean ± SD.

## Data Availability

The data presented in this study are available on request from the corresponding author.
